# A Fault Diagnosis Method Considering Meteorological Factors for Transmission Networks Based on P Systems

**DOI:** 10.3390/e23081008

**Published:** 2021-08-01

**Authors:** Xiaotian Chen, Tao Wang, Ruixuan Ying, Zhibo Cao

**Affiliations:** 1 School of Electrical Engineering and Electronic Information, Xihua University, Chengdu 610039, China; txc0226@163.com (X.C.); yingruixuan123@gmail.com (R.Y.); CaoZhiBo2021@163.com (Z.C.); 2 Key Laboratory of Fluid and Power Machinery, Ministry of Education, Xihua University, Chengdu 610039, China; 3 Key Laboratory of Fluid Machinery and Engineering, Sichuan Province, Xihua University, Chengdu 610039, China

**Keywords:** spiking neural P system, fault diagnosis, meteorological factor, gray fuzzy

## Abstract

Bad meteorological conditions may reduce the reliability of power communication equipment, which can increase the distortion possibility of fault information in the communication process, hence raising its uncertainty and incompleteness. To address the issue, this paper proposes a fault diagnosis method for transmission networks considering meteorological factors. Firstly, a spiking neural P system considering a meteorological living environment and its matrix reasoning algorithm are designed. Secondly, based on the topology structure of the target power transmission network and the action logic of its protection devices, a diagnosis model based on the spiking neural P system considering the meteorological living environment is built for each suspicious fault transmission line. Following this, the action messages of protection devices and corresponding temporal order information are used to obtain initial pulse values of input neurons of the diagnosis model, which are then modified with the gray fuzzy theory. Finally, the matrix reasoning algorithm of each model is executed in a parallel manner to obtain diagnosis results. Experiment results achieved out on IEEE 39-bus system show the feasibility and effectiveness of the proposed method.

## 1. Introduction

As the core part of a power system, the power transmission network has always provided the important guarantee of power transmission and distribution. Consequently, the safe and stable operation of a transmission network is crucial to the electrical industry. However, impacted by various factors, such as weather interruption and system errors, failures of transmission networks may occur [1,2].

Therefore, several fault diagnosis approaches of power systems for the aided decision making have been developed, such as expert systems [3], Bayesian networks [4], the rough set theory [5], artificial neural networks [6], Petri nets [7,8,9], cause–effect networks [10,11], the fuzzy theory [12], and spiking neural P systems (SNPSs) [13,14,15,16,17]. Among above methods, the SNPS is a class of distributed parallel computing models based on structures and functions of nerve cells. Due to its powerful ability in information processing and parallel computing (most of the models have been proved to be Turing equivalent [18]), the SNPS has become a hot research topic in the field of membrane computing and fault diagnosis. To date, each kind of methods has its own merits and application scenarios. However, existing studies do not consider the impact of meteorology factors on the diagnosis accuracy of the methods.

Unfortunately, in recent years, faults of transmission lines frequently occur due to bad meteorological conditions. For example, Ref. [19] classified fault data of 110 kV transmission lines for the power grid of Guangxi province in China between 2012 and 2014. The related results showed that the external meteorological environment of transmission lines was the main cause of their faults, accounting for up to 78.09%. Moreover, [20] showed that unplanned outage events caused by natural disasters and meteorological factors account for 84.36% of all the unplanned outages of 220–500 kV overhead transmission lines.

It can be seen that the external meteorological environment of transmission networks is a kind of impact factor that cannot be ignored due to their faults. Usually, under meteorological conditions, such as the thunder and lightning, typhoons, ice, and forest fires, the fault rate of transmission lines will increase. Furthermore, these bad conditions can also easily reduce the reliability of power communication equipment, hence increasing the distortion possibility of fault information. Consequently, the accuracy of fault diagnosis methods based on fault indicators [21,22] or regional communication data [23,24] will decrease. Therefore, it is necessary to the consider meteorological environment as an essential information source when developing fault diagnosis methods for transmission networks.

To solve the above-mentioned issues, this paper proposes a spiking neural P system considering a meteorological living environment (mleSNPS) for the fault diagnosis of transmission networks in the framework of membrane computing. The main contributions of this paper are described as follows:

(1) To deal with the uncertainty of fault information that is caused by its distortion possibility under bad meteorological conditions, this paper synthetically utilizes the action messages and temporal order information of protection devices as well as the meteorological information of target transmission networks;
(2) To effectively handle the meteorological information, the mleSNPS and its matrix reasoning algorithm are proposed. In the mleSNPS, the meteorological information is processed by the meteorological living environment of input neurons so as to correct their initial pulse values;
(3) To improve diagnostic accuracy, a fault diagnosis method based on the mleSNPS and reasoning algorithm is proposed. An mleSNPS-based diagnosis model for each suspicious fault transmission line is built. The reasoning algorithms of all the models are executed in a parallel manner to obtain diagnostic results. The experimental results show that the proposed method is feasible and effective.

The remainder of this paper is organized as follows: Section 2 proposes the mleSNPS and its reasoning algorithm. In Section 3, the mleSNPS-based fault diagnosis method is presented. After that, Section 4 applies the proposed method to an IEEE 39-bus system to show its feasibility and effectiveness. Finally, conclusions are drawn in Section 5.

## 2. Spiking Neural P System Considering Meteorological Living Environment

### 2.1. MleSNPS

**Definition** **1.** *A spiking neural P system considering the meteorological living environment (mleSNPS) with a degree of m≥1 is a tuple*

$$\Pi \;=\;(O,\sigma_{1},\ldots ,\sigma_{m},syn,in,out,\kappa )$$

*where*

*(1)*  *O={a} is a set of singleton alphabets, and a denotes a*spike.
*(2)*  *$\sigma_{1},\ldots ,\sigma_{m}$ are neurons, which consist of two classes, i.e., rule neurons and proposition neurons. A rule neuron corresponds to a fuzzy fault production rule, while a proposition neuron is associated with a proposition in the rule. Each neuron $\sigma_{i}$(i=1,…,m) is of the form $\left(\theta_{i},c_{i},{\vec{\omega }}_{i},\lambda_{i},r_{i}\right)$, where*
  *(a)*  *$\theta_{i}$ is a real number in [0,1], which denotes the pulse value of the neuron.*
  *(b)*  *$c_{i}$ is a real number in [0,1], which represents the fuzzy truth value of the neuron. If $\sigma_{i}$ is a proposition neuron, then $c_{i}=0$; otherwise, $c_{i}$ is equal to the certainty factor of the fuzzy production rule corresponding to $\sigma_{i}$;*
  *(c)*  *${\vec{\omega }}_{i}=\left(\omega_{1\to i},\ldots ,\omega_{N_{i}\to i}\right)$ expresses the input weight vector of $\sigma_{i}$, where $\omega_{k\to i}\;(k\;=\;1,\ldots ,N_{i})$ is set as 0.5 or 1, representing the weight value of $\sigma_{i}$ from its k-th presynaptic neuron; $N_{i}$ is a natural number, denoting the number of synapses that end at neuron $\sigma_{i}$. Specifically, input weights beginning from input neurons are set as 0.5, while the others are set as 1. This is because an input neuron corresponds to a protection device (i.e., a protective relay or a circuit breaker). Usually, the two kinds of protection devices play an equally important role in computation.*
  *(d)*  *$\lambda_{i}$ is a real number in [0,1], which indicates the firing (spiking) threshold of the neuron. In this paper, $\lambda_{i}=0.2$ [13];*
  *(e)*  *$r_{i}$ represents the firing rule of $\sigma_{i}$, which is in the form of $E/a^{\theta }\to a^{\beta }$, where both θ and β are real numbers in [0,1]; $E=\{a^{n},\;\theta \geq \lambda_{i}\}$ denotes the firing condition of $r_{i}$. The fire rule can be applied if and only if it receives at least n spikes with potential value $\theta \geq \lambda_{i}$. When different types of neurons execute the firing rules, their pulse values are updated in different manners, which is explained in detail following Definition 1.*
*(3)*  *syn⊆{1,…,m}×{1,…,m} denotes the directional-weighted synaptic connection between neurons in Π, where i≠j for all (i,j)∈syn(1≤i,j≤m).*
*(4)*  *in,out⊆{1,…,m} represent the sets of input and output neurons, respectively. It is worth noting that an input neuron corresponds to a protection device (i.e., a protective relay or a circuit breaker) of a suspicious fault transmission line. Initial pulse values of input neurons represent the correction values of fusion results of action information of corresponding protection devices and temporal order information. An output neuron is associated with a suspicious fault transmission line, and its pulse value denotes the fault confidence level of the corresponding section.*
*(5)*  *κ=(γ,ξ,f,ρ) indicates the meteorological living environment of input neurons, where*
  *(a)*  *γ is a real number in [0,1] representing the failure risk value of a suspicious transmission line considering meteorological factors.*
  *(b)*  *ξ is a real number in (0,1) representing the weight of γ in input parameters of a fault diagnosis model. Its value is set according to the influence degree of a meteorological level on the communication system of a power grid.*
  *(c)*  *f denotes the firing threshold of an eliminating rule of the meteorological living environment. In this paper, f is set as 0.5 according to expertise.*
  *(d)*  *ρ expresses an eliminating rule, whose firing condition is E={γ<f}, meaning that rule ρ can be applied if and only if γ<f. Afterwards, the influence of meteorological factors on the transmission line will not be considered in the diagnosis model; i.e., the influence of meteorological factors on a line fault will not be taken into account in the subsequent diagnosis process. Otherwise, the rule ρ cannot be executed. In this case, the influence of meteorological factors on the fault should be considered in the diagnosis process; that is, the fault risk value γ should be one of the input parameters of the diagnosis model.*

An mleSNPS system contains one kind of proposition neurons and three types of rule neurons (i.e., “general”, “and”, and “or” rule neurons). After executing $E/a^{\theta }\to a^{\beta }$ in $\sigma_{i}$, the refresh scheme of pulse values in each type of neurons are described as follows.

(1) If a proposition neuron $\sigma_{i}$ is not an input neuron, then $\beta =\max(\theta_{1}*\omega_{1},\ldots ,\theta_{k}*\omega_{k})$, where $\theta_{i}*\omega_{i}\;(1\leq$i≤k) represents the weighted pulse value of the *i*-th presynaptic neuron of $\sigma_{i}$.

(2) A “general” rule neuron $\sigma_{i}$ has only one presynaptic neuron, corresponding to a simple fault fuzzy production rule. It is denoted by the symbol “General”. For this kind of neuron, β=θ*ω*c, where θ*ω denotes the weighted pulse value of the presynaptic neuron of $\sigma_{i}$ and *c* indicates the certainty factor of the fault rule.

(3) An “and” rule neuron $\sigma_{i}$ has k(k≥2) presynaptic neurons, corresponding to a compound “and” fuzzy fault production rule. It is denoted by the symbol “And”. For this kind of neuron, $\beta =[\left(\theta_{1}*\omega_{1}+\ldots +\theta_{k}*\omega_{k}\right)/\left(\omega_{1}+\ldots +\omega_{k}\right)]*c$, where $\theta_{i}*\omega_{i}\;\left(1\leq i\leq k\right)$ denotes the weighted pulse value of the *i*-th presynaptic neuron of $\sigma_{i}$ and *c* denotes the certainty factor of the fault rule.

(4) An “or” rule neuron $\sigma_{i}$ has k(k≥2) presynaptic neurons, corresponding to a compound “or” fuzzy fault production rule. It is denoted by the symbol “Or”. For this kind of neuron, $\beta =max\{\theta_{1}*\omega_{1},\ldots ,\theta_{k}*\omega_{k}\}*c$, where $\theta_{i}*\omega_{i}\;\left(1\leq i\leq k\right)$ represents the weighted pulse value of the *i*-th presynaptic neuron of $\sigma_{i}$ and *c* denotes the certainty factor of the fault rule.

**Figure 1:**
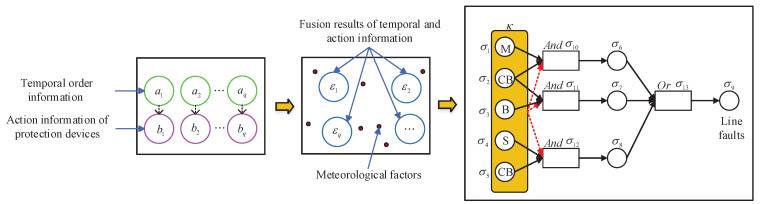
Sketch map of building process of mleSNPS-based diagnosis model.

To improve the intelligibility, a sketch map of the building process of an mleSNPS-based diagnosis model is shown in Figure 1, where black circles represent proposition neurons, black rectangles indicate rule neurons, and yellow rounded rectangles express the meteorological living environment; black solid arrows indicate weighted synapses between neurons, and red dotted arrows denote influence weights of meteorological living environment on rule neurons.

The statistics in [25] showed that many meteorological factors have adverse effects on diagnostic accuracy, such as thunder and lightning, typhoon, snow, wind, ice, rainfall, and hail. In this paper, fault risk levels of transmission lines are firstly evaluated by considering the above seven typical meteorological factors. An evaluation value is the fault rate of each meteorological factor at a meteorological level [26], as shown in Table 1, where each fault rate is calculated by dividing the number of faults occurring on a transmission line under a meteorological factor level in one year into the occurrence number of the meteorological level in the same year. The four colors (i.e., blue, yellow, orange, and red) in Table 1 represent four meteorological levels in China, which are “general”, “heavier”, “severe”, and “particularly” serious, respectively.

After that, the information in Table 1 is processed using a gray fuzzy comprehensive evaluation model [27,28]. In this paper, the risk levels of different meteorological factors for transmission lines are divided into four types, i.e., $G=\{G_{1},G_{2},G_{3},G_{4}\}$, which are associated with levels of “low risk”, “general risk”, “comparative high risk”, and “high risk”, respectively. The corresponding evaluation criteria for these risk levels are shown in Table 2. Consequently, failure risk values can be obtained based on the risk levels, and these are used as input parameters in mleSNPS-based diagnosis models. The method for obtaining failure risk values is described in detail in Section 3, step (3)-(b)-(v).

### 2.2. Matrix Reasoning Algorithm

In order to enable an mleSNPS to infer and process fault information in a parallel manner, this paper devises Algorithm 1.
**Algorithm 1:** Matrix reasoning algorithm.**Input:**$\theta_{0},\delta_{0},\gamma ,\xi ,f,\eta ,C,W_{r1},W_{r2},W_{r3},W_{p},\lambda_{p},\lambda_{r}$  **Step (1)**: set termination conditions: $0_{1}={\left\{0,\ldots ,0\right\}}_{d}$, $0_{2}={\left\{0,\ldots ,0\right\}}_{l}$;**Step (2)**: let g=0, where *g* is the reasoning step;**Step (3)**: **while**$\theta_{g}\neq 0_{1}$ or $\delta_{g}\neq 0_{2}$ **do****Step (4)**: **for** each input neuron (g=0) or each proposition neuron (g>0) **do**;**Step (5)**: evaluate firing conditions of meteorological living environment and each proposition neuron. **If**E={γ<f} is not satisfied and $E=\{a^{n},\theta_{i}\geq \lambda_{pi},1\leq i\leq d\}$ is satisfied, **then** compute the pulse value vector of rule neurons $\delta_{g+1}$ via performing $\delta_{g+1}=\left[\left(\theta_{g}⊗W_{r1}\right)+\left(\theta_{g}⊕W_{r2}\right)+\left(\theta_{g}⊙W_{r3}\right)\right]•\eta +(\gamma ⊗\xi )$;**Step (6)**: **else if** both E={γ<f} and $E=\{a^{n},\theta_{i}\geq \lambda_{pi},1\leq i\leq d\}$ are satisfied, **then** compute the pulse value vector of rule neurons $\delta_{g+1}$ via performing $\delta_{g+1}=\left(\theta_{g}⊗W_{r1}\right)+\left(\theta_{g}⊕W_{r2}\right)+\left(\theta_{g}⊙W_{r3}\right)$;**Step (7)**: **end if****Step (8)**: **if** the input neuron or proposition neuron has postsynaptic rule neurons, **then** it will send a new spike to its postsynaptic neurons;**Step (9)**: **else** it just accumulates pulse values;**Step (10)**: **end if****Step (11)**: **end for****Step (12)**: **for** each rule neuron **do**;**Step (13)**: evaluate the firing condition of each rule neuron. **If**$E=\{a^{n},\delta_{j}\geq \lambda_{rj},1\leq j\leq l\}$ is satisfied, **then** compute the pulse value vector of proposition neurons $\theta_{g+1}$ via performing $\theta_{g+1}=\left(\delta_{g}⊗C\right)⊙W_{p}$;**Step (14)**: **end if****Step (16)**: **end for****Step (15)**: g=g+1.**Step (17)**: **end while****Output:** pulse values of output neurons

To make the algorithm more intuitive, vectors and matrices involved in it are explained as follows:

(1) $\theta =(\theta_{1},\ldots ,\theta_{d})$ denotes the pulse value vector of proposition neurons, where $\theta_{i}\;\left(1\leq i\leq d\right)$ is a real number in [0,1].
(2) $\delta =(\delta_{1},\ldots ,\delta_{l})$ denotes the pulse value vector of rule neurons, where $\delta_{j}\;\left(1\leq j\leq l\right)$ is a real number in [0,1].
(3) $\lambda_{p}=\left(\lambda_{p1},\ldots ,\lambda_{pd}\right)$ and $\lambda_{r}=\left(\lambda_{r1},\ldots ,\lambda_{rl}\right)$ denote firing threshold vectors of proposition and rule neurons, respectively. They consist of firing thresholds of *d* proposition neurons and *l* rule neurons, respectively, where d+l=m, and both $\lambda_{pi}$ and $\lambda_{rj}$ are real numbers in [0,1).
(4) γ is a real number in [0,1], denoting the failure risk value of a suspicious transmission line considering the impact of meteorological factors.
(5) $\xi =(\xi_{1},\ldots ,\xi_{l})$ denotes the directed connection vector of meteorological living environment to rule neurons. If there is such a connection, then $\xi_{j}\in \left(0,1\right)$ represents the weight of a failure risk value in the input parameters of an mleSNPS-based diagnosis model; otherwise, $\xi_{j}=0$.
(6) $\eta ={\left(\eta_{ij}\right)}_{d\times l}$ denotes the directed connection matrix from input neurons to rule neurons. If an input neuron $\sigma_{i}$ connects a rule neuron $\sigma_{j}$, then $\eta_{ij}\in \left(0,1\right)$ represents its weight value to the rule neuron; otherwise, $\eta_{ij}=1$.
(7) $C=diag(c_{1},\ldots ,c_{l})$ denotes the true value matrix of rule neurons, where $c_{j}\;\left(1\leq j\leq l\right)$ is a real number in [0,1] representing the certainty factor of rule neuron $\sigma_{j}$.
(8) $W_{r1}={\left(\omega_{ij}\right)}_{d\times l}$, $W_{r2}={\left(\omega_{ij}\right)}_{d\times l}$, and $W_{r3}={\left(\omega_{ij}\right)}_{d\times l}$ denote directed synaptic connection matrices from a proposition neuron $\sigma_{i}$ to a “general”, “and”, and “or” rule neuron $\sigma_{j}$, respectively. If there is such a connection, then $\omega_{ij}\in \left(0,1\right]$ represents the output weight value of proposition neuron $\sigma_{i}$ to rule neuron $\sigma_{j}$; otherwise, $\omega_{ij}=0$.
(9) $W_{p}={\left(\omega_{ji}\right)}_{l\times d}$ denotes the directed synaptic connection matrix from a rule neuron $\sigma_{j}$ to a proposition neuron $\sigma_{i}$. If there is such a connection, then $\omega_{ji}\in \left(0,1\right]$ represents the output weight value of rule neuron $\sigma_{j}$ to proposition neuron $\sigma_{i}$; otherwise, $\omega_{ji}=0$.

Next, the operators are introduced as follows:

(1) $\theta ⊗W_{r}=\left(\psi_{1},\ldots ,\psi_{l}\right)$, where $\psi_{j}=\theta_{1}*\omega_{1j}+\ldots +\theta_{d}*\omega_{dj}$, j=1,…,l.

(2) $\theta ⊕W_{r}=\left(\psi_{1},\ldots ,\psi_{l}\right)$, where $\psi_{j}=\left(\theta_{1}*\omega_{1j}+\ldots +\theta_{d}*\omega_{dj}\right)/\left(\omega_{1j}+\ldots +\omega_{dj}\right)$, j=1,…,l.

(3) $\gamma ⊗\xi =(\psi_{1},\ldots ,\psi_{l})$, where $\psi_{j}=\gamma *\xi_{j}$, j=1,…,l.

(4) $\delta •\eta =(\psi_{1},\ldots ,\psi_{l})$, where $\psi_{j}=\delta_{j}*\left[(\eta_{1j}+\ldots +\eta_{dj})/d\right]$, j=1,…,l.

(5) $\theta ⊙W_{r}=\left(\psi_{1},\ldots ,\psi_{l}\right)$, where $\psi_{j}=\max\left(\theta_{1}*\omega_{1j},\ldots ,\theta_{d}*\omega_{dj}\right)$, j=1,…,l. Similarly, $\delta ⊙W_{p}=\left(\psi_{1},\ldots ,\psi_{d}\right)$, where $\psi_{i}=\max\left(\delta_{1}*\omega_{1i},\ldots ,\delta_{l}*\omega_{li}\right)$, i=1,…,d.

## 3. Fault Diagnosis Method of Transmission Networks Considering Meteorological Factors

**Figure 2:**
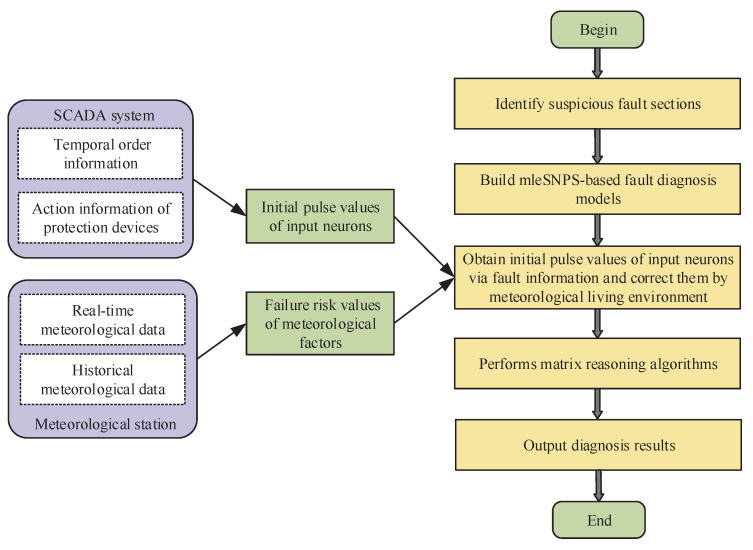
Flowchart of proposed method.

This section proposes a fault diagnosis method for transmission networks based on mleSNPSs considering meteorological factors, whose flowchart is shown in Figure 2.

Detailed steps of the proposed method are described as follows:

Step (1): Identify suspicious fault transmission lines. Determine fault areas via the network topology-based analysis method so as to identify suspicious fault transmission lines.

Step (2): Build an mleSNPS-based fault diagnosis model for each suspicious fault transmission line based on the topology structure of target power transmission network and the action logic of its protection devices.

Step (3): Obtain initial pulse values of input neurons for each diagnosis model via fault information and correct them via the meteorological living environment.

**Figure 3:**
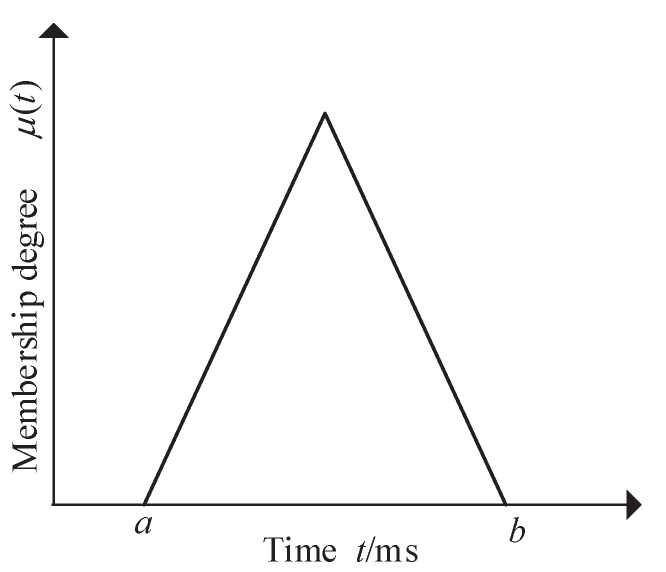
A triangle membership function.

(a) For each diagnosis model, obtain initial pulse values of input neurons. The three steps are described as follows:

(i) Obtain action messages of protection devices from the supervisory control and data acquisition (SCADA) system. Then, obtain the operation or non-operation confidence levels [16] of these devices according to Table 3 and Table 4.

(ii) Calculate membership values of the action moment of protection devices by fuzzing fault temporal order information via Equation (1), whose corresponding triangle membership function is shown in Figure 3.

(1) $$\mu \left(t\right)=\left\{\begin{array}{c} 0,t\leq a,t\geq b\\ (t-a)/((b-a)/2),a<t\leq (a+b)/2\\ (b-t)/((b-a)/2),(a+b)/2<t<b \end{array}\right.$$

where *t* denotes the moment that a protective relay operates or a circuit breaker trips; μ(t) represents the membership value corresponding to *t*; *a* and *b* indicate lower and upper bounds of the time delay of protective devices, respectively. The time delay [9] used in this paper includes the following: $D\left(t_{f},t_{M}\right)=\left[10,40\right]$ ms, $D\left(t_{f},t_{B}\right)=\left[300,500\right]$ ms, $D\left(t_{f},t_{S}\right)=\left[600,1000\right]$ ms, and $D\left(t_{R},t_{CB}\right)=\left[40,60\right]$ ms, where $D(t_{f},t_{M})$, $D(t_{f},t_{B})$, and $D(t_{f},t_{S})$ represent the time delay between the moment of a line faults and the action moment of main protections, first backup protections, and second backup protections, respectively; $D(t_{R},t_{CB})$ denotes the time delay between the action moment of protective relays and the corresponding circuit breakers.

(iii) Obtain the initial pulse values of input neurons. Perform fusion calculations via Equation (2) for operation confidence levels (or non-operation confidence levels) of the protection devices and membership values of their action moment.

(2) $$\mu \left(\sigma_{i}\right)=1-\left(1-\mu \left(t_{i}\right)\right)\left(1-\mu \left(v_{i}\right)\right)$$

where $\mu (\sigma_{i})$ denotes the pulse value of the *i*-th input neuron; $\mu (t_{i})$ indicates the membership value of action moment of the protection device corresponding to the *i*-th input neuron; $\mu (v_{i})$ represents the operation confidence level (or non-operation confidence level) of the protection device corresponding to the *i*-th input neuron.

**Figure 4:**
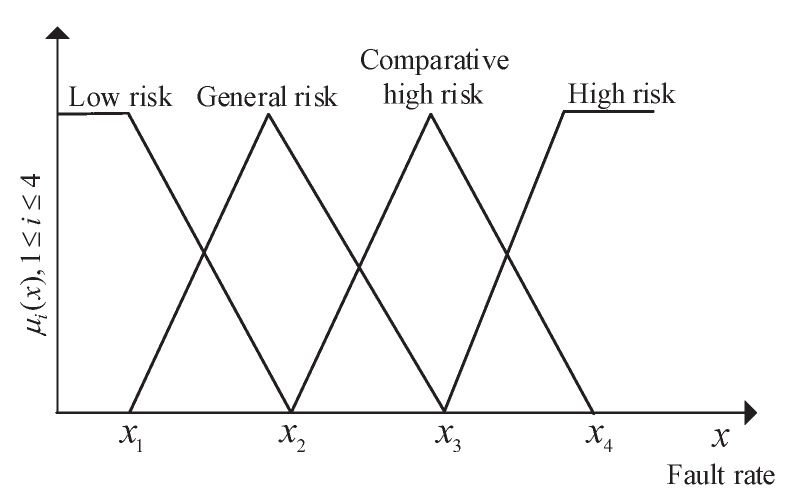
Triangle membership function of meteorological factors.

(b) Correct the obtained initial pulse values using the meteorological living environment.

(i) Obtain real-time meteorological data of the fault areas from meteorological stations. Then, obtain the fault rates corresponding to meteorological factors at the fault moment according to Table 1.

(ii) Construct a gray fuzzy membership matrix for involved meteorological factors. Firstly, based on Table 2 and Figure 4, the fault rates are brought into Equations (3)–(6) to calculate membership values $\mu_{i}\;\left(1\leq i\leq p\right)$ corresponding to different risk levels of relevant meteorological factors, where *p* is the number of risk levels. Secondly, judge the completeness of meteorological information and assign its corresponding level with the gray scale $u_{i}$. Finally, according to $\mu_{i}$ and $u_{i}$, obtain the gray fuzzy membership matrix $\underset{⊗}{\tilde{R}}\;={\left[\left(\mu_{ij},u_{ij}\right)\right]}_{n\times p}$ of the meteorological factors, where *n* denotes the number of meteorological factors.

(3) $$\mu_{1}\left(x\right)=\left\{\begin{array}{c} 0,x\geq x_{2}\\ \left(x_{2}-x\right)/\left(x_{2}-x_{1}\right),x_{1}\leq x<x_{2}\\ 1,x<x_{1} \end{array}\right.$$

(4) $$\mu_{2}\left(x\right)=\left\{\begin{array}{c} \left(x_{3}-x\right)/\left(x_{3}-x_{2}\right),x_{2}\leq x<x_{3}\\ 0,x\geq x_{3},x\leq x_{1}\\ \left(x-x_{1}\right)/\left(x_{2}-x_{1}\right),x_{1}<x<x_{2} \end{array}\right.$$

(5) $$\mu_{3}\left(x\right)=\left\{\begin{array}{c} \left(x_{4}-x\right)/\left(x_{4}-x_{3}\right),x_{3}\leq x<x_{4}\\ 0,x\geq x_{4},x\leq x_{2}\\ \left(x-x_{2}\right)/\left(x_{3}-x_{2}\right),x_{2}<x<x_{3} \end{array}\right.$$

(6) $$\mu_{4}\left(x\right)=\left\{\begin{array}{c} 1,x\geq x_{4}\\ \left(x-x_{3}\right)/\left(x_{4}-x_{3}\right),x_{3}\leq x<x_{4}\\ 0,x<x_{3} \end{array}\right.$$

where $x_{1},x_{2},x_{3}$, and $x_{4}$ denote boundary values of $G_{1},G_{2},G_{3}$, and $G_{4}$, respectively [29].

(iii) Construct a weight vector for meteorological factors. Firstly, according to Table 5, establish a relationship matrix between every two meteorological factors, i.e., $A\;=\;{\left(a_{ij}\right)}_{n\times n}$(1≤i,j≤n), where $a_{ij}$ denotes the importance of *i*-th meteorological factor to the *j*-th one, and *n* represents the number of meteorological factors involved. Secondly, calculate the weight value $w_{i}$ for each meteorological factor based on matrix A. Thirdly, judge the relevance between the *i*-th meteorological factor and the residual ones, and then assign the *i*-th factor with gray scale $h_{i}$. Finally, obtain the weight vector for meteorological factors based on $w_{i}$ and $h_{i}$, i.e., $\underset{⊗}{\tilde{W}}\;={\left[\left(w_{i},h_{i}\right)\right]}_{n\times 1}^{T}\;\left(1\leq i\leq n\right)$, where $w_{i}$ is calculated by Equation (7).

(7) $$w_{i}=\frac{\sqrt[{n}]{\prod_{j=1}^{n}a_{ij}}}{\sum_{i=1}^{n}\sqrt[{n}]{\prod_{j=1}^{n}a_{ij}}}$$

(iv) Obtain risk levels of suspicious transmission lines. According to Equation (8), calculate a gray fuzzy evaluation vector of meteorological factors, i.e., $\underset{⊗}{\tilde{B}}$. Then, obtain risk levels of suspicious transmission lines based on the maximum membership principle and minimum gray scale principle.

(8) $$\underset{⊗}{\tilde{B}}\;=\underset{⊗}{\tilde{W}}\;\cdot \underset{⊗}{\tilde{R}}\;=\left[{\left(\sum_{i=1}^{n}w_{i}*\mu_{ij},\prod_{i=1}^{n}1\wedge (h_{i}+u_{ij})\right)}_{1\times 4}\right]$$

(v) Calculate the failure risk value for each suspicious transmission line considering meteorological factors based on the obtained risk levels and data in Table 6.

(vi) Correct the initial pulse values of the input neurons for the mleSNPS-based diagnosis model using the obtained failure risk values.

Step (4): Each mleSNPS-based diagnosis model performs its matrix reasoning algorithm in a parallel manner to calculate pulse values of its output neurons.

Step (5): Output diagnosis results. Determine fault lines according to the pulse values of the output neurons. If the pulse value of an output neuron is greater than 0.5, then its corresponding transmission line has a fault; otherwise, it is not faulty.

## 4. Experimental Results and Analysis

**Figure 5:**
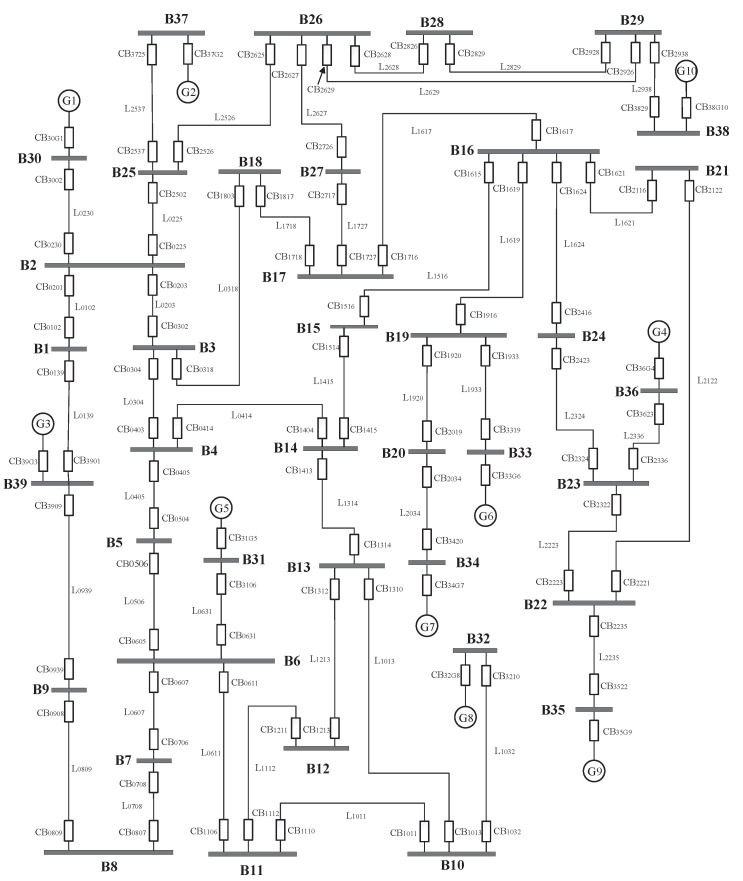
IEEE 39-bus system.

In this section, case studies and accurate tests based on the IEEE 39-bus system, as shown in Figure 5, are used to verify the feasibility and effectiveness of the proposed method. In the real world, it is rare that all meteorological factors appear simultaneously. Therefore, for both the case study and accuracy test, this section uses a common combination of meteorological factors according to actual climate conditions in a certain region, including thunder and lightning (orange), wind (blue), and rainfall (yellow), as well as the levels of the other meteorological factors, which are equal to 1.

### 4.1. Case Study

Five cases under above meteorological factors are considered, whose diagnosis results are shown in Table 7. From the table, we can see that for cases 1–4, all the three methods can find the right fault transmission lines. For case 5, only the proposed method can diagnose the right transmission lines, while both the method in [15] and that in [30] only find one fault line. Moreover, the diagnosis results of our method also include fault confidence levels of fault lines and evaluation of fault information. Therefore, the proposed method is effective with a good accuracy in diagnosing faults of transmission lines.

Next, case 1 is taken as an example to illustrate how the proposed method works.

Fault information of case 1: operated relays and tripped CBs: $\left(SLR_{1803},0\right)$ ms, $\left(SLR_{0403},2\right)$ ms, $\left(SLR_{0203},6\right)$ ms, $\left(CB_{1803},48\right)$ ms, $\left(CB_{0403},53\right)$ ms and $\left(CB_{0203},56\right)$ ms; protective relays and CBs refuse to operate: ${BR}_{03}$, ${CB}_{0302}$, ${CB}_{0304}$ and ${CB}_{0318}$. Meteorological information of case 1: thunder and lightning (orange), wind (blue), rainfall (yellow), and levels of other meteorological factors are equal to 1.

**Figure 6:**
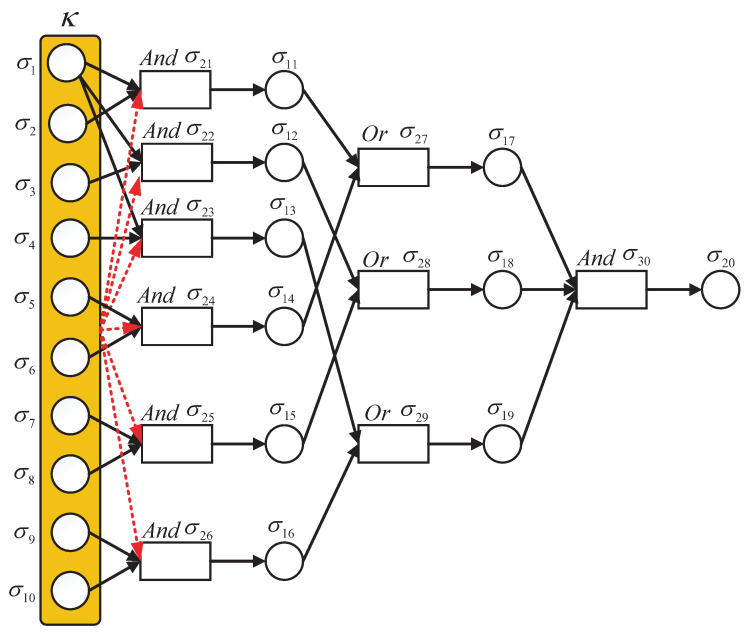
The mleSNPS-based fault diagnosis model of $B_{03}$.

The mleSNPS-based fault diagnosis model of $B_{03}$ is built as shown in Figure 6.

According to the fault information, we obtain the initial pulse value vector of input neurons.

$$\theta_{0}=\left[0.2,0.2,0.2,0.2,0.7,0.95,0.7,0.925,0.7,0.9,0_{1\times 10}\right]$$

Based on the meteorological information of case 1 and data in Table 1, we note that fault rates under thunder and lightning, typhoon, snow, wind, ice, rainfall, and hail are 0.70, 0.002, 0.003, 0.12, 0.0032, 0.08, and 0.0028, respectively.

Based on the above fault rate, and data in Table 2 and Figure 4, we make calculations to obtain the gray fuzzy membership matrix of meteorological factors, i.e., $\underset{⊗}{\tilde{R}}\;$.

$$\underset{⊗}{\tilde{R}}\;=\left[\begin{array}{cccc} \left(0,0.2\right) & \left(0,0.4\right) & \left(0,0.3\right) & \left(1,0.2\right)\\ \left(1,0\right) & \left(0,0.2\right) & \left(0,0.3\right) & \left(0,0.1\right)\\ \left(1,0.2\right) & \left(0,0\right) & \left(0,0.2\right) & \left(0,0.3\right)\\ \left(0,0.3\right) & \left(0,0.2\right) & \left(0.57,0.3\right) & \left(0.43,0.2\right)\\ \left(0.99,0.2\right) & \left(0.01,0.2\right) & \left(0,0.2\right) & \left(0,0.3\right)\\ \left(0,0.2\right) & \left(0,0.3\right) & \left(0,0.3\right) & \left(1,0.2\right)\\ \left(0.9,0.2\right) & \left(0.1,0.2\right) & \left(0,0.2\right) & \left(0,0.3\right) \end{array}\right]$$

Then, according to Table 5, we construct the relationship matrix between every two meteorological factors, i.e., A.

$$A=\left[\begin{array}{ccccccc} 1 & 6 & 7 & 3 & 7 & 3 & 5\\ 1/6 & 1 & 4 & 1/6 & 3 & 1/3 & 1/3\\ 1/7 & 1/4 & 1 & 1/5 & 1/2 & 1/6 & 1/4\\ 1/3 & 6 & 6 & 1 & 6 & 2 & 5\\ 1/7 & 1/3 & 2 & 1/6 & 1 & 1/5 & 1/2\\ 1/3 & 3 & 6 & 1/2 & 5 & 1 & 4\\ 1/5 & 3 & 4 & 1/5 & 2 & 1/4 & 1 \end{array}\right]$$

ion matrix A, we obtain the weight vector of meteorological factors, i.e., $\underset{⊗}{\tilde{W}}\;$.

$$\underset{⊗}{\tilde{W}}\;={\left[\begin{array}{c} \left(0.377,0.3\right)\\ \left(0.0607,0.3\right)\\ \left(0.0276,0.3\right)\\ \left(0.2423,0.3\right)\\ \left(0.0387,0.3\right)\\ \left(0.1744,0.3\right)\\ \left(0.0793,0.3\right) \end{array}\right]}^{T}$$

The gray fuzzy evaluation vector of meteorological factors, i.e., $\underset{⊗}{\tilde{B}}\;$, is calculated via Equation (8).

$$\underset{⊗}{\tilde{B}}\;={\left[\begin{array}{c} \left(0.198,0.0113\right)\\ \left(0.0083,0.0263\right)\\ \left(0.1381,0.0162\right)\\ \left(0.6556,0.0108\right) \end{array}\right]}^{T}$$

From the evaluation of $\underset{⊗}{\tilde{B}}\;$ via the maximum membership principle and minimum gray scale principle, we note that bus $B_{03}$ is in the high risk area. Consequently, combined with the data in Table 6, we further note that the fault risk value of the bus $B_{03}$ is equal to 1, i.e., $\gamma_{B_{03}}=1$.

Then, the matrix reasoning algorithm is performed to obtain pulse values of output neurons of the diagnosis model of bus $B_{03}$.

when g=0, we obtain the results

$$\delta_{1}=\left[0.52,0.52,0.52,0.895,0.8575,0.88,0_{1\times 4}\right];$$

$$\theta_{1}=\left[0_{1\times 10},0.507,0.507,0.507,0.8055,0.7718,0.792,0_{1\times 4}\right].$$

when g=1, we obtain the results

$$\delta_{2}=\left[0_{1\times 6},0.8055,0.7718,0.792,0\right];$$

$$\theta_{2}=\left[0_{1\times 16},0.7854,0.7525,0.7722,0\right].$$

when g=2, we obtain the results

$$\delta_{3}=\left[0_{1\times 9},0.77\right];$$

$$\theta_{3}=\left[0_{1\times 19},0.7506\right].$$

when g=3, we obtain the results

$$\delta_{4}=\left[0_{1\times 10}\right].$$

Consequently, the termination condition is satisfied and the reasoning process ends. The output neuron exports its pulse value, i.e., 0.7506, denoting bus $B_{03}$ has a fault with a fault confidence level 0.7506.

### 4.2. Accuracy Test

To verify the fault-tolerant ability and universality of the proposed method, we conduct an accuracy test under a different uncertain information ratio.

(9) $$\varepsilon =\frac{I_{\mathrm{random}}}{I_{fault}}\times 100\%$$

where ε represents the uncertain information ratio; $I_{\mathrm{random}}$ is the number of uncertain fault alarm messages; and $I_{fault}$ is the number of fault alarm messages got from the SCADA system. It is noteworthy that an information ratio is obtained by randomly mixing uncertain information into the right fault information, where the uncertain one is obtained by simulating refusing and unwanted operations of protection devices as well as information loss and distortion in the signal transmission process.

To ensure the universality of experimental results, each diagnosis accuracy in Table 8 is obtained based on the IEEE-39 bus system by testing them one million times. Moreover, this table also shows the comparison results of our method with two baseline ones from [15,30]. From Table 8, we can see that when ε=0 (i.e., all fault messages are correct), the accuracies of all three methods are 100%. However, due to the introduction of meteorological factors, the proposed method has the highest accuracy when the value of ε rises.

## 5. Conclusions

To improve the diagnosis accuracy of transmission lines, this paper proposes a fault diagnosis method considering meteorological factors. It can effectively utilize three kinds of information, i.e., action messages of protective devices, the temporal order information in the SCADA system, and the meteorological information of target transmission networks. To achieve this goal, the mleSNPS and corresponding matrix reasoning algorithm are proposed, where the meteorological living environment is introduced to process the meteorological information. Due to the comprehensive utilization of different kinds of information and introduction of the meteorological living environment, the diagnostic accuracy of the proposed method increases. Comparison results show its feasibility and effectiveness. This paper only studies the impact of meteorological factors on the uncertainty of fault alarm information. In the future, we will further study fault diagnosis and recovery methods of power systems for extreme climate disasters.

## Figures and Tables

**Table 1:** Fault rates of seven typical meteorological factors under different levels.

Thunder and Lightning	Red (0.84)	Orange (0.7)	Yellow (0.52)	Level 2 (0.05)	Level 1 (0.0038)
Typhoon	Red (0.68)	Orange (0.53)	Yellow (0.35)	Blue (0.13)	Level 6 (0.09)
	Level 5 (0.07)	Level 4 (0.01)	Level 3 (0.005)	Level 2 (0.0035)	Level 1 (0.002)
Snow	Red (0.65)	Orange (0.35)	Level 3 (0.045)	Level 2 (0.008)	Level 1 (0.003)
Wind	Red (0.64)	Orange (0.44)	Yellow (0.24)	Blue (0.12)	Level 6 (0.08)
	Level 5 (0.06)	Level 4 (0.008)	Level 3 (0.0043)	Level 2 (0.0031)	Level 1 (0.0014)
Ice	Red (0.62)	Orange (0.44)	Yellow (0.35)	Level 2 (0.04)	Level 1 (0.0032)
Rainfall	Red (0.42)	Orange (0.21)	Yellow (0.08)	Rainstorm (0.05)	Heavy rain (0.03)
	Moderate rain (0.0038)	Light rain (0.001)	−	−	−
Hail	Red (0.34)	Orange (0.12)	Level 3 (0.035)	Level 2 (0.0033)	Level 1 (0.0028)

**Table 2:** Evaluation criteria for risk levels of different meteorological factors for transmission lines.

Meteorological Factors	Risk Levels			
	$G_{1}$	$G_{2}$	$G_{3}$	$G_{4}$
Thunder and lightning	≤0.05	0.003∼0.06	0.05∼0.5	≥0.06
Typhoon	≤0.06	0.003∼0.07	0.06∼0.1	≥0.07
Snow	≤0.04	0.003∼0.06	0.04∼0.1	≥0.06
Wind	≤0.05	0.002∼0.06	0.05∼0.2	≥0.06
Ice	≤0.03	0.003∼0.04	0.03∼0.3	≥0.04
Rainfall	≤0.01	0.002∼0.02	0.01∼0.05	≥0.02
Hail	≤0.01	0.002∼0.02	0.01∼0.08	≥0.02

**Table 3:** Operation confidence levels for protection devices.

Sections	Main		First Backup		Second Backup	
	Protections		Protections		Protections	
	Protective Relays	CBs	Protective Relays	CBs	Protective Relays	CBs
Bus	0.8564	0.9833	-	-	0.7	0.75
Line	0.9913	0.9833	0.8	0.85	0.7	0.75

**Table 4:** Non-operation confidence levels for protection devices.

Sections	Main		First Backup		Second Backup	
	Protections		Protections		Protections	
	Protective Relays	CBs	Protective Relays	CBs	Protective Relays	CBs
Bus	0.2	0.2	-	-	0.2	0.2
Line	0.2	0.2	0.2	0.2	0.2	0.2

**Table 5:** Important levels between every two meteorological factors.

Numbers	Important Levels
1	Equally important
3	Slightly important
5	Obviously important
7	Strongly important
9	Extremely important
2, 4, 6, 8	Between above levels

**Table 6:** Failure risk values of transmission lines corresponding to different risk levels of meteorological factors.

Risk Levels	Failure Risk Values
$G_{1}$	0.25
$G_{2}$	0.5
$G_{3}$	0.75
$G_{4}$	1

**Table 7:** Comparisons of diagnosis results between the proposed method and two baseline fault diagnosis methods.

Cases	Fault Information		Actual			The Proposed Method		
	Protective	Circuit	Fault	Ref. [15]	Ref. [30]	Fault Confidence	Diagnosis	Information
	Relays	Breakers	Lines			Levels	Results	Evaluation
1	$\left(SLR_{1803},0\right)$	$\left(CB_{1803},48\right)$						
	$\left(SLR_{0403},2\right)$	$\left(CB_{0403},53\right)$	$B_{03}$	$B_{03}$	$B_{03}$	0.7506	$B_{03}$	Correct action
	$\left(SLR_{0203},6\right)$	$\left(CB_{0203},56\right)$						
2	$\left(BR_{18},0\right)$	$\left(CB_{1817},35\right)$						Correct action
	$\left(MLR_{1817},120\right)$	$\left(CB_{1803},40\right)$	$B_{18}$	$B_{18}$	$B_{18}$	0.8823	$B_{18}$	
	$\left(MLR_{1718},128\right)$	$\left(CB_{1718},143\right)$	$L_{1718}$	$L_{1718}$	$L_{1718}$	0.8990	$L_{1718}$	
		$\left(CB_{1817},167\right)$						
3		$\left(CB_{0302},49\right)$	$B_{03}$	$B_{03}$	$B_{03}$	0.8342	$B_{03}$	
	$\left(BR_{03},0\right)$	$\left(CB_{0304},51\right)$						Error message:
	$\left(SLR_{1803},650\right)$	$\left(CB_{1803},650\right)$						$\left(CB_{0203},700\right)$
		$\left(CB_{0203},700\right)$						
4		$\left(CB_{0302},38\right)$						
	$\left(BR_{03},0\right)$	$\left(CB_{0304},42\right)$						
	$\left(BR_{14},100\right)$	$\left(CB_{1404},295\right)$	$B_{03}$	$B_{03}$	$B_{03}$	0.8266	$B_{03}$	Error message:
	$\left(SLR_{1803},598\right)$	$\left(CB_{1415},298\right)$	$B_{14}$	$B_{14}$	$B_{14}$	0.8843	$B_{14}$	$\left(CB_{1413},385\right)$
		$\left(CB_{1413},385\right)$						
		$\left(CB_{1803},623\right)$						
5		$\left(CB_{1817},35\right)$						Miss message:
	$\left(BR_{18},0\right)$	$\left(CB_{1803},40\right)$	$B_{18}$	$B_{18}$	$B_{18}$	0.8823	$B_{18}$	$\left(MLR_{0318},-\right)$
	$\left(MLR_{1803},100\right)$	$\left(CB_{1718},96\right)$	$L_{0318}$			0.7900	$L_{0318}$	Error message:
		$\left(CB_{0318},135\right)$						$\left(CB_{1718},96\right)$

**Table 8:** Accuracy tests of three methods for different ε.

ε	mleSNPS	FRSNPS [15]	Petri Net [30]
0	100%	100%	100%
1	99.91%	99.18%	99.77%
2	99.83%	98.36%	99.54%
3	99.74%	97.54%	99.31%
4	99.65%	96.72%	99.08%
5	99.57%	95.90%	98.85%
6	99.48%	95.08%	98.62%
7	99.39%	94.26%	98.39%
8	99.31%	93.44%	98.16%
9	99.22%	92.62%	97.92%
10	99.13%	91.79%	97.69%
20	98.26%	83.59%	95.39%
30	97.39%	75.38%	93.08%

## Data Availability

Not applicable.
